# Comparing Behavioral-Emotional Difficulties in Siblings of Children with and without Sensory Impairments

**Published:** 2019-03

**Authors:** Setareh SHOJAEE, Hamid ALIZADEH

**Affiliations:** 1. Department of Special Education, Faculty of Education and Psychology, Shiraz University, Shiraz, Iran; 2. Department of Special Education, Faculty of Psychology and Education, Allameh Tabataba’i University, Tehran, Iran

**Keywords:** Emotional/behavioral difficulties, Siblings, Children, Sensory impairments

## Abstract

**Background:**

Siblings of children with disabilities are the most likely family members to be affected by the child’s disability due to the long-lasting nature of relationships between siblings compared to those of other family members. The aim of the present study was to compare the emotional-behavioral difficulties (EBD) in siblings of children with and without Sensory Impairments (SI).

**Methods:**

The statistical population of this causal-comparative research included all siblings of children with and without SI in Shiraz, southern Iran in 2016. The sample consisted of ninety-one siblings of children with (38) and without (53) SI in Shiraz. Sample of siblings of children with SI was recruited by purposeful sampling and sample of siblings of children without sensory impairments were selected through a multistage random sampling method. Strengths and Difficulties Questionnaire (SDQ) was utilized for measuring EBD. Two way ANOVA and MANOVA tests were used for data analyses.

**Results:**

Total EBD and two subscales of EBD (emotional problems and peer relationship problems) in siblings of children with SI were significantly greater than the comparison group (*P*<0.001). Furthermore, no significant difference was observed in the two subscales (conduct problems, and hyperactivity) between these two groups.

**Conclusion:**

This study provides strong evidence that siblings of children with SI are significantly at a higher risk of psychological problems, and accordingly we suggest for the related supports and services.

## Introduction

According to family systems perspective, the problems of any member of family affect other family members and subsystems such as siblings’ relations (1). One of the factors affecting the personal and social lives of family members is the disability of one family member (2). The disabled person’s siblings are more affected due to their longest relations with each other among other family members (3); their behavioral-emotional development is affected the most (4). For instance, these siblings experience various challenges such as observing the disabled child’s physical and emotional problems (5), mental health problems (6), low self-concept, depression, behavioral problems (7), being neglected (8), and more stress (9). In other words, these siblings, facing their disabled sister or brother, experience conflicting emotions such as fear of death of the disabled sibling, jealousy, anger, loneliness, and isolation (5). The healthy siblings of the disabled children experience substantial problems such as behavioral-emotional ones due to other family members’ inattention, limited opportunities to express negative emotions and feelings, withdrawal from their peers, and inadequate information about the disability of their sibling (10). These problems, if not studied and relieved or reduced by intervention or prevention, will continue, worsen (11), and put the healthy siblings in danger of developing psychological disorders (6). In line with the studies on the necessary interventional and preventive measures, scientific evidence suggests that emotional problems such as depression and anxiety in the disabled children’s siblings are more than those in siblings of typically-developing children (12). Moreover, the feelings of shame, fear, isolation, guilt, irritability, aggression, and conflict with peers are more in these siblings compared to typically-developing siblings (13).

Although the majority of the research shows that disabled children’s siblings experience more severe emotional-behavioral difficulties (14–17), there are several other studies pointing out no difference in their emotional-behavioral difficulties (18–22).

However, based on the present researchers’ study, there is no research on the conditions of the emotional-behavioral difficulties of the siblings of children with sensory impairment and since emotional-behavioral difficulties affect all aspects of personal and social lives of the siblings of the children with sensory impairments, Therefore, insufficient attention to their emotional-behavioral difficulties can make them more serious and chronic. Considering the importance of these problems, every attempt to detect, diagnose, prevent, control, and treat of emotional-behavioral difficulties will be valuable and result in the promotion of the society’s mental health and In this regard, comparing the emotional-behavioral difficulties of the siblings of the children with sensory impairments and those of siblings with typically-developing brother or sister is a valuable strategy for detecting and diagnosing emotional-behavioral difficulties as well as drafting comprehensive preventive and interventional plans. Moreover, this research can provide suitable information on emotional-behavioral difficulties of these siblings for parents, educators, schools and decision-making centers for mental health programs and activities.

For this reason, the aim of the present study was to compare the emotional-behavioral difficulties (EBD) in siblings of children with and without Sensory Impairments (SI) and was executed to answer the following question: Is there a gender-specific significant difference between the siblings of the children with sensory impairment and the siblings of the children without sensory impairment with regard to emotional-behavioral difficulties?

## Methods

### Participants and Procedure

The statistical population comprised all siblings (10–18 yr of age) of the children with or without sensory impairments in Shiraz, southern Iran in 2016. The samples were 91 teenagers (38 people were the siblings of children with sensory impairments and 53 were the siblings of those without sensory impairments) that siblings of children with sensory impairments were recruited through convenience sampling method and siblings of children without sensory impairments were selected by multistage random sampling method.

The Ethical Review Boards of the regional Regular Education Organization, the regional Special Education Organization and the regional Welfare Organization approved the study.

Sample characteristics for siblings of children with and without sensory impairments are presented in Table 1. There were no significant differences in the mean age, ratio of boys to girls, birth order, family size, maternal educational level, and family income between the two groups (Table 1).

**Table 1:** Sample characteristics for siblings of children with and without Sensory Impairments

***Variable***	***siblings of children with sensory impairments (N = 38)***	***siblings of children without sensory impairments(N = 53)***
Mean age (yr) (SD)	14 (2.73)	14.55 (2.66)
Range (yr)	10–18	10–18
Male (Female)	14 (24)	17 (36)
Birth order (SD)	1.74 (0.63)	2 (1.03)
Family size (SD)	4.88 (0.94)	4.73 (1.03)
Maternal educational level (%): < 12 yr (> 12 yr)	83.94 (16.06)	77.36 (22.64)
Family income (%): (≤10,000,000 IRR, 10,000,001–30,000,000 IRR, ≥30,000,001 IRR)	(68.42, 28.95, 2.63)	(66.04, 30.19, 3.77)

US$1 = 39200 IRR.

### Measures: The Strengths and Difficulties Questionnaire (SDQ)

The Strengths and Difficulties Questionnaire (SDQ) (23) was used in the study. It features 25 items and 5 subscales of emotional symptoms, conduct problems, hyperactivity, peer relationship problems, and prosocial behavior. The sum of the first 4 subscales is the emotional-behavioral difficulties score. The questionnaire is a three-point Likert scale, including incorrect (zero), correct to some extent (score 1), and quite correct (score 2).

A higher score indicates more emotional-behavioral difficulties. The SDQ is a valid tool for measuring children’s behavioral adjustment (24). In identifying the behavioral problems of children, it is as efficient as the Achenbach’ behavior checklist and Rutter scales (23). Previous research using the SDQ for siblings of children with developmental disabilities, autism spectrum disorder and intellectual disability has reported appropriate reliability and validity (15). The self-rated SDQ has satisfactory discriminability between community and clinical samples (25). In the present study, the internal consistency in the self-reported SDQ for the total difficulties scores was .72.

### Procedure

Initially, a license was obtained from the regional Regular Education Organization, regional Special Education Organization, and regional Welfare Organization of Shiraz. Then, by referring to schools and special centers for people with sensory impairments under the supervision of the regional Special Education Organization and the regional Welfare Organization of Shiraz, all children with sensory impairment that had siblings from 10 to 18 yr old were selected. The siblings of these children were asked to participate in the study if they wanted to participate. In this way, 38 completed questionnaires were collected from siblings of children with sensory impairment. In addition, for the selection of siblings of children without sensory impairment, the list of schools in Shiraz was prepared and randomly four girls’ schools (two high schools and two elementary schools) and four male schools (two high schools and two elementary schools) were selected. By referring to these schools, students who had siblings from 10 to 18 yr old were randomly selected and their siblings were asked to participate in the study if they wanted to participate. In this way, 53 completed questionnaires were collected from siblings of children without sensory impairment.

### Statistical analysis

The data were analyzed using the SPSS software (version 19.0 (Chicago, IL, USA). After checking the data for normal distribution, the parametric procedures were used to analyze them. A 2-way analysis of variance and multivariate analysis of variance (MANOVA) was used to assess the statistical significance of the effect of group and gender as the independent variable on the dependent variables.

## Results

In this section, first, the mean (SD) scores of subjects’ emotional-behavioral difficulties and their subscales with respect to their group and gender have been provided.

As is shown in Table 2, the mean score of the emotional-behavioral difficulties of the siblings of children with sensory impairments is higher than that of the siblings of children without sensory impairments. Then, in order to test the significance of the hypothesis, we used 2-way analysis of variance which its results are presented in Table 3. Before performing 2-way ANOVA, the Levene’s test was done to assess the assumption of homogeneity of the variances, which was not significant (Levene’s test =0.768). Therefore, the use of 2-way ANOVA was approved.

**Table 2:** Mean and standard deviation of scores for emotional-behavioral difficulties of subjects according to group and gender

***Variable***	***Group***	***Siblings of children with sensory impairments***		***Siblings of children without sensory impairments***	
***Dependent variables***	***Gender***	***Mean***	***SD***	***Mean***	***SD***
Total score of emotional-behavioral difficulties	Female	15.59	6.26	9.52	5.61
	Male	15.13	4.46	11.23	5.58
	Total	15.39	5.51	10.36	5.61
Emotional symptoms	Female	4.73	2.31	2.56	2.34
	Male	4.38	2.42	2.38	2.29
	Total	4.59	2.33	2.47	2.30
Conduct problems	Female	2.50	1.37	1.89	1.69
	Male	2.44	1.21	2.42	1.47
	Total	2.47	1.29	2.15	1.60
Hyperactivity	Female	3.55	2.26	2.74	1.81
	Male	3.63	1.89	3.92	2.65
	Total	3.58	2.09	3.32	2.33
Peer relationship problems	Female	4.82	2.28	2.33	1.44
	Male	4.69	1.78	2.50	1.39
	Total	4.76	2.06	2.42	1.41

**Table 3:** Results of two-way analysis of variance between groups for siblings of children with and without sensory impairments

***Source of changes***	***Sum of squares***	***df***	***Mean of squares***	***F***	***P***
Group	541.47	1	541.47	17.33	0.001
Gender	8.47	1	8.47	0/604	0.003
Interaction between group and gender	25.86	1	25.86	0.83	0.37
Error	2718.43	87	31.25		
Total	17452	91			

Between groups 2-way ANOVA shows that the effects are significant for groups [F (1, 87) =17.33, *P*<0.001, ***η2***=0.17], but for the subjects’ genders [F (1, 112) =2.455, ***η2***=0.003] and group interaction with subjects’ genders [F (1, 112) =1.315, ***η2***=0.009], are not significant. In addition to compare the subscales of emotional-behavioral difficulties among the siblings of children with and without sensory impairments, multivariate analysis of variance (MANOVA) was employed and its results are shown in Table 4. Prior to MANOVA, to assess the assumption of homogeneity of the variances, Levene’s test was performed. This test was not significant for any subscale. Consequently, using MANOVA was permitted. Also to examine the homogeneity of covariance matrices, Box’s M Test was administered the Box’s M statistics were not significant at 47.773 [F=1.45, *P*>0.05], and therefore the assumption of the covariance homogeneity is met. Based on the data presented in Table 4, the effect of group on linear combinations of dependent variables is significant. That is why an ANOVA was run to assess the significance of this effect on each dependent variable (Table 5).

**Table 4:** Wilks lambda values in multivariate analysis of variance for subscales of emotional/behavioral problems

***Wilks’ lambda Source***	***Value***	***F***	***df hypothesis***	***Df error***	***P***
Group	0.650	11.33	4	84	0.001
Gender	0.963	0.814	4	84	0.52
Interaction between group and gender	0.980	0.434	4	84	0.78

**Table 5:** Results of Multivariate analysis of variance for dependent variables

***Variable***	***Group***		***Gender***		***Interaction between age group and gender***	
***Dependent variable***	***F***	***P***	***F***	***P***	***F***	***P***
Emotional symptoms	17.29	0.001	0.273	0.602	0.033	0.857
Conduct problems	0.974	0.326	0.554	0.459	0.887	0.349
Hyperactivity	0.288	0.593	1.788	0.185	1.366	0.249
Peer relationship problems	39.94	0.001	0.002	0.961	0.162	0.689

The impact of group on the dependent variables of emotional symptoms [F=17.29, *P*<0.001] and peer relationship problems [F=39.94, *P*<0.001] are significant. The average score of the siblings of children with sensory impairments in the above-mentioned variables was higher than that in the siblings of children without sensory impairments. The effects of group on the variables of conduct problems [F=0.974] and hyperactivity [F=0.288] are not significant. In all subscales, the effects of the subjects’ gender and interaction between group and subjects’ gender were not significant.

## Discussion

The findings indicated that emotional-behavioral difficulties were significantly more in the siblings of children with sensory impairments in comparison with the siblings of typically-developing children. More problems in siblings of children with sensory impairments can be due to the attitude and characteristics of healthy siblings. These healthy siblings may not feel to be adequately supported by the family and consider the disabled child as a factor of negligence of the family (26). This attitude, in turn, may worsen their emotional-behavioral difficulties. These siblings should take more caring roles compared to typically-developing siblings (27, 28), resulting in putting them under mental pressure and worsen their emotional-behavioral difficulties.

In addition, more emotional-behavioral difficulties in siblings of disabled children may be due to parenting style. In this regard, parenting style plays a role in emotional-behavioral difficulties (29). Moreover, more emotional-behavioral difficulties in siblings of children with sensory impairments can result from the disabled person’s characteristics. Likewise, the problems of disabled children would negatively affect other siblings’ adjustment (15).

The comparison of emotional-behavioral difficulties’ subscales among two groups (siblings of children with sensory impairments and siblings of children without sensory impairments) showed that the emotional problems of the siblings of children with sensory impairments are more than that of the siblings of children without sensory impairments. Consistent with this finding, 30.5% of healthy siblings of the children with disability had significant emotional-behavioral symptoms and these symptoms have a negative and significant correlation with their quality of life (6). In addition, 50% of the healthy siblings of disabled children have emotional problems which in turn would lead to their more negative moods (30). Elaborating this, these siblings experience a high degree of anxiety, depression, aggression, withdrawal, and restlessness (31); often find themselves lonely due to weak relations among family members (32); have constant worries about responsibilities of taking care of disabled children in the future (33); and in general, have more psychological problems and less intimate relations (34, 35). All together these challenges may result in increased emotional problems in the siblings of children with sensory impairments.

In addition, research findings showed the poor peer relation skills in the siblings of children with sensory impairments. To explain these findings, one can assert that peer relation is mostly the result of participation in corporate activities with peers and taking part in extracurricular activities (36), while these siblings are expected to take more caring responsibilities and housework (37). They spend most of the time with their families and contribute to the management of the disabled child’s affairs (38). Therefore, they have less time for leisure activities and interacting with friends and society, and as a result, they will not properly learn peer relation skills and so have more problems in their peer relations.

Moreover, negative behavior that people with sensory impairments display (39) also affects the peer relations of their siblings. Moreover, peer relationship problems may be due to the fact that healthy siblings are ashamed of others knowing about their disabled sibling or do not know how to answer questions of their friends about their disabled sibling and how to explain the situation (26). Another reason why these healthy siblings have peer relation problems is that parents make them take their disabled sibling when hanging out with their peers, and engage them in their social activities (40). This may sometimes be rejected by the peers which cause problems in peer relations. In this study, no significant difference was observed between siblings of children with and without sensory impairments with regard to hyperactivity and conduct problems. To explain this finding, clinically significant hyperactivity and conduct problems have been seen in a few siblings of disabled children (41). Perhaps that is why no significant difference was detected between two groups in terms of hyperactivity and conduct problems. Furthermore, neurological and genetic predispositions are the major factors in people’s getting hyperactivity and conduct problems (42). This factor can also justify this lack of difference between groups with regard to hyperactivity and conduct problems.

In the end, this research has been done on the siblings of children with and without sensory impairments, so that the results can only be generalized to this society. Considering the age limit of the present study, conducted only in the age group of 10 to 18 yr old, the generalization of its results to other age groups requires more research. Future researchers are recommended to work on other disabilities and also on the relation between emotional-behavioral difficulties of the siblings of children with sensory impairments and demographic variables such as parental education, parental age, parental occupation, parental income, number of family members, and the birth order of the child with sensory problem. Given the vulnerability of the siblings of the children with sensory impairments with respect to emotional-behavioral difficulties, preparing and designing supportive plans to reduce their emotional-behavioral difficulties is essential. Moreover, interventions to increase knowledge and awareness of the families about the effects of the child’s disabilities on their healthy siblings will be useful.

## Conclusion

Emotional-behavioral difficulties in siblings of children with sensory impairments were significantly greater than those of siblings of typically developing children. Moreover, in subscales of emotional-behavioral difficulties, siblings of children with sensory impairments scored significantly higher than siblings of typically developing children in emotional symptoms and peer relationships. Furthermore, no significant difference was found in hyperactivity and conduct problems between study groups.

Due to the potentially negative effects of having a sibling with sensory impairments, it is important to continue the research on this topic and to identify support and strategies that could promote positive outcomes and adjustment for typically developing siblings of children with sensory impairment.

## Ethical considerations

Ethical issues (Including plagiarism, informed consent, misconduct, data fabrication and/or falsification, double publication and/or submission, redundancy, etc.) have been completely observed by the authors.
